# Prognostic Value of *LINC-ROR* (rs1942347) Variant in Patients with Colon Cancer Harboring BRAF Mutation: A Propensity Score-Matched Analysis

**DOI:** 10.3390/biom12040569

**Published:** 2022-04-12

**Authors:** Aly A. M. Shaalan, Sara H. Mokhtar, Hanadi Talal Ahmedah, Amany I. Almars, Eman A. Toraih, Afaf T. Ibrahiem, Manal S. Fawzy, Mai A. Salem

**Affiliations:** 1 Department of Anatomy, Faculty of Medicine, Jazan University, Jazan 45142, Saudi Arabia; ashaalan@jazanu.edu.sa; 2 Department of Histology, Faculty of Medicine, Suez Canal University, Ismailia 41522, Egypt; 3 Department of Medical Laboratory Sciences, Faculty of Applied Medical Sciences, King Abdulaziz University, Jeddah 21589, Saudi Arabia; amokhtar@kau.edu.sa (S.H.M.); aialmars@kau.edu.sa (A.I.A.); 4 Department of Medical Laboratory Technology, Faculty of Applied Medical Sciences, King Abdulaziz University, Rabigh 21911, Saudi Arabia; hehmedouh@kau.edu.sa; 5 Center of Innovation in Personalized Medicine (CIPM), King Abdulaziz University, Jeddah 21589, Saudi Arabia; 6 Department of Surgery, Division of Endocrine and Oncologic Surgery, School of Medicine, Tulane University, New Orleans, LA 70112, USA; 7 Genetics Unit, Department of Histology and Cell Biology, Suez Canal University, Ismailia 41522, Egypt; 8 Department of Pathology, Faculty of Medicine, Northern Border University, Arar 1321, Saudi Arabia; afaf.taha@nbu.edu.sa; 9 Department of Pathology, Faculty of Medicine, Mansoura University, Mansoura 35516, Egypt; 10 Department of Medical Biochemistry and Molecular Biology, Faculty of Medicine, Suez Canal University, Ismailia 41522, Egypt; 11 Department of Biochemistry, Faculty of Medicine, Northern Border University, Arar 1321, Saudi Arabia; 12 Department of Medical Biochemistry, Faculty of Medicine, Mansoura University, Mansoura 35516, Egypt; maisalem@mans.edu.eg

**Keywords:** colon cancer, LINC-ROR, rs1942347, single nucleotide polymorphism, prognosis

## Abstract

Emerging studies show that long intergenic non-protein coding RNA, regulator of reprogramming (*LINC-ROR*) is aberrantly expressed in several types of cancer, including colon cancer (CC). *LINC-ROR* intronic variant rs1942347 may impact gene regulation and disease phenotype. We aimed to explore the potential association of *LINC-ROR* (rs1942347) with the clinicopathological features and outcome of CC cases. Archived FFPE (n = 180) CC samples were enrolled. Taq-Man allelic discrimination PCR was used for genotyping in propensity-matched cohorts with/without positive staining for mutant BRAF protein after eliminating confounders bias. The rs1942347*A allele variant was associated with high pathological grade, larger tumor size, distant metastasis, and mortality. Multiple logistic regression analysis adjusted by sex and BRAF mutation showed A/A genotype carriers to have 3 times more risk of early onset of cancer (OR = 3.13, 95%CI = 1.28–7.69, *p* = 0.034) than T/T genotype carriers. Overall analysis showed that rs1942347*A allele carriers had higher risk of mortality under heterozygote (OR = 2.13, 95%CI = 1.08–4.35, *p* = 0.003), homozygote (OR = 5.0, 95%CI = 1.69–14.29, *p* = 0.003), dominant (OR = 3.33, 95%CI = 1.20–9.09, *p* = 0.003), and recessive (OR = 2.63, 95%CI = 1.37–5.0, *p* = 0.011) models compared to T/T allele carriers. Stratified analysis by BRAF status revealed that the ancestor T/T allele conferred protection in BRAF mutant CC patients and was associated with a 73–93% reduced risk of mortality under heterozygote/homozygote comparison models. Using Kaplan–Meier curves, carriers of the A/A genotype had shorter survival times than T/T cohorts. The univariate Cox regression model revealed that the A/A genotype was associated with a 3.5 times greater mortality risk than the T/T genotype. However, after adjustment by multiple Cox regression analysis, the risk was insignificant. In conclusion, this is the first study identifying the potential association of the *LINC-ROR* (rs1942347) variant with CC prognosis.

## 1. Introduction

Colon cancer (CC) remains one of the most common lethal malignant tumors worldwide, with a steadily rising incidence rate in developing countries [1,2]. The global new colorectal cancer cases is predicted to reach 3.2 million in 2040 [3]. Although several screening/prognostic markers and therapeutic modalities for CC have been identified, many patients, in particular, in developing countries, are diagnosed at late stages with tumor invasion/distance metastasis [4].

Accumulating evidence indicates that colon tumorigenesis is a multistage process in which the contribution of environmental, genetic, and/or epigenetic factors is evident [5,6,7]. Recently, the long non-coding RNAs (lncRNAs) family of non-coding RNAs have been identified as one of the main contributors to CC tumorigenesis and could have potential novel diagnostic/prognostic utility in CC [5,8,9,10] As a new intergenic ncRNA, the long intergenic noncoding-regulator of reprogramming; *LINC-ROR* has been identified as a key player in the development, progression, epithelial to mesenchymal transition (EMT), and invasion/metastasis of multiple cancers, including CC [11,12,13,14,15]. The related gene (ID: 100885779) is located on Ch. 18q21.31 (the reverse strand), spanning about 17.5 Kb (Ensembl.org). It consists of 6 exons, coding for two transcripts of 2603 and 2278 nucleotide bases in length (https://www.ncbi.nlm.nih.gov/gene/100885779, accessed on 10 February 2022).

Numerous studies have reported that dysregulated expression of *LINC-ROR* in CC contributes to cancer cell viability, proliferation, invasion, and/or metastasis and functions as competing endogenous RNA by sponging microRNA-145 and miRNA-223-3p or regulating the miR-6833-3p/SMC4 pathway [10,14,16]. Furthermore, *LINC-ROR* could also promote radiotherapy resistance in human colorectal cells by targeting the p53/miR-145 pathway [17].

Although the studies mentioned above unraveled the impact of aberrant *LINC-ROR* expression on CC development and progression, the clinical importance of the specified *LINC-ROR* gene variant(s) in CC remains largely unknown. Accumulating evidence revealed that lncRNAs single nucleotide polymorphisms (SNPs) could significantly impact the lncRNA secondary structure, expression levels, and/or processing, which results in tumorigenesis and progression and drug response alteration [5,18].

Across the *LINC-ROR*-related SNPs detected during genome sequencing of different ethnic groups, only five intronic variants were cited (Supplementary Table S1). These include rs1942348 (T/C), rs6420545 (C/T), rs9636089 (C/T), and rs4801078 (C/T) in breast cancer [19] and rs732982 (G/A) in schizophrenia [20]. We selected the intronic variant rs1942347 (A/T), which overlaps 3 out of 8 transcripts of the gene (i.e., *LINC-ROR*-202, 207, and 208), has the highest minor allele frequency (MAF; 0.47) among the identified related variants, according to the HapMap project, and was not cited before. In this sense, the authors were interested in exploring the prognostic value of this variant for the first time, to our knowledge, in patients with colon cancer using TaqMan Real-Time allelic discrimination PCR. In association with other genetic, epigenetic, and environmental markers, this could be helpful in future personalized management for patients with CC.

## 2. Materials and Methods

### 2.1. Patients and Tissues

A total of 351 paraffin-embedded blocks of CC tissue specimens were collected from Suez Canal University hospital pathology lab, Ismailia, Oncology Center of Mansoura Hospital, Mansoura, and El-laban Pathology Lab, Port-Said, Egypt, in the last 5 years, and complete clinical and pathological data were screened. Of these, 60 patients had positive staining for mutant BRAF protein (BRAF*^V600E^*). Propensity scores matching analysis yielded 2 similar datasets of 60 and 120 matched cohorts with mutant and wild-type BRAF tumors, respectively. Inclusion criteria included the presence of sufficient tissue specimens for pathological and molecular work. Exclusion criteria were receiving any treatment modality before surgery, secondary tumors, loss of follow-up, missing clinical and/or pathological data, samples without paired non-cancer tissues, and samples with insufficient quality of extracted DNA. The demographic data, such as the patient’s age, sex, tumor location, and postoperative course (recurrence and survival), were obtained from the patients’ medical records. The International Union Against Cancer TNM staging system [21] was applied for the cancer staging system. The Declaration of Helsinki ethical guidelines were followed. The local Medical Research Ethics Committee approved this study. The patient consent was waived as the enrolled samples in this retrospective study were archived.

### 2.2. Histopathological and Immunohistochemical Assessment

A total of 351 samples were included initially at the start of the analysis. Serial sections of 5 μm from each paraffin-embedded block of tumor tissue specimens were stained with H&E. Revision of the histopathological features of each tumor was reviewed by an expert histopathologist as regards the variants (adenocarcinoma, mucinous, and signet ring), the differentiation, and the presence of lymphovascular invasion according to WHO classification [22]. TNM staging and Dukes’ staging of each tumor were reviewed according to Akkoca and colleagues [23]. The paraffin tissue sections were dewaxed, rehydrated, and washed in phosphate-buffered saline 1× (PBS; Lonza, Verviers, Belgium). Antigen retrieval was performed by treating the slides in a PT Link (Dako, Agilent Technologies, Santa Clara, CA, United States) containing acid or basic solution (as appropriate) and preheated to 97 °C for 30 min. Next, the tissue was treated with a peroxidase-blocking solution (Dako, Agilent Technologies, Santa Clara, CA, United States) for five minutes. Mouse monoclonal antibodies were applied: anti-BRAF VE1 (Catalog No. ab228461, dilution 1:100, Abcam, Waltham, MA, United States). The sections were counterstained for three minutes with Meyer’s hematoxylin, and then mounted. Human melanoma tissue with B-RAF V600E mutation tissues were run as a positive control as recommended by the manufacturer. Negative controls were obtained by omitting the primary antibodies.

### 2.3. Interpretation of the Immunohistochemical Results

All immune-stained slides were evaluated two times by the pathologist, blinded to all clinical, histopathological, and genetic data. The CC cases were scored positive for BRAF V600E mutation if ≥80% of tumor cells expressed diffuse uniform unequivocal strong or moderate cytoplasmic staining with or without nuclear staining. In contrast, the cases were negative for BRAF V600E mutation if they showed no staining or weak, cytoplasmic, non-granular, uniform staining (stain intensity < 80%). Cases with staining of isolated cells in a tumor and those who showed no staining were also negative. The cases were scored as equivocal if they displayed ambiguous, heterogeneous, non-uniform cytoplasmic staining in tumor cells with or without nuclear staining [24,25,26].

It is worth noting that cases that showed equivocal results of IHC staining underwent molecular screening and had *BRAF* mutation results defined as positive or negative documented in their medical record.

### 2.4. In Silico Data Analysis

Genomic structure and variants of *LINC-ROR* were identified in the Ensembl Genomic database (www.ensembl.org). The list was sorted, and the most common biallelic variant rs1942347 (A/T) was selected. The putative variant effect was explored in the HaploReg v4.1 database (http://compbio.mit.edu/HaploReg) (accessed on 10 February 2021) to investigate the presence of linked SNPs and small indels within the block. Prior publications were retrieved from the human gene database GeneCards (www.genecards.org) and the NCBI (https://www.ncbi.nlm.nih.gov/) (all databases last accessed on 10 February 2021) [27].

### 2.5. *LINC-ROR* rs1942347 (A/T) Variant Analysis

Tissue genomic DNA of 180 CC samples was extracted and purified according to the QIAamp DNA FFPE Tissue Kit protocol (Catalog no. 56404, Qiagen, Hilden, Germany), which depends on the selective binding of DNA to the silica-based membranes after tissue digestion by proteinase K and incubation at an elevated temperature (90 °C) to reverses the formalin crosslinking. RNase was added during the extraction procedure to obtain RNA-free genomic DNA. The isolated DNA was quantified using a Nanodrop-1000 spectrophotometer (NanoDrop Tech., Wilmington, USA) and stored at − 80 °C for the time of allelic discrimination analysis. The specified variant was genotyped using a TaqMan assay (Cat no. C__11450075_10) with specific probe-fluorescence dyes to detect the transversion substitution A/T of interest in the context sequence [VIC/FAM]GGTGTATACCTAGGAGCAAAGTTGC[A/T]GGGTCATATGGGAACCCTATGTTTA] according to the Chr.18: 57057227 on build GRCh38. The Real-Time PCR was performed by two independent coauthors blinded to the BRAF status of the samples in a StepOne™ Real-Time PCR System (Applied Biosystems, Foster City, CA, USA) as detailed in our previous publications [28,29]. Nuclease-free water was loaded instead of the extracted DNA in each run to work as non-template negative controls. The PCR set was programmed to run the initial denaturation step at 95 °C for 10 min, followed by 40 cycles of amplification at 95 °C for 15 s and annealing at 60 °C for 1 min, and the final step at 60 °C (30 s). In total, 10% of the samples were randomly assigned for a rerun to ensure reproducibility of the results with a 100% recalling genotyping rate. SDS software version 1.3.1. (Applied Biosystems) was applied for genotyping data analysis.

### 2.6. Statistical Analysis

Statistical analysis was carried out using SPSS v27.0 (IBM Corp.) and R software version 3.4.2 (R Foundation). Propensity score analysis was performed using the nearest neighbor method with a ratio of 2:1 using the MatchIT R package. Hardy–Weinberg analysis was computed through SNPstats software [30]. Allele and genotype frequencies were estimated as previously described [31]. The odds ratio (OR) and 95% confidence intervals (CIs) were calculated for each inheritance association model [31]. The Akaike information criterion (AIC) was used to compare different possible models and determine which best fitted the data. A two-sided Chi-square test was applied to compare clinical and pathological data between groups. Kaplan–Meier survival curves were reconstructed to analyze the survival times for each specified genotype carrier. A univariate Cox regression model followed by multiple Cox regression analysis was run. The 2-sided *p*-value was significant at <0.05.

## 3. Results

### 3.1. Characteristics of the Study Population

The study included 180 colon cancer patients: 120 patients with wild-type BRAF protein and 60 patients in the propensities-matched BRAF mutation group. There was no significant difference between the two groups regarding their demographic, clinical, and pathological features (Supplementary Table S2). Those patients who died were more likely to have tumors in the transverse or descending colon (61.3% vs. 38.1%, *p* = 0.004), high pathological grade (37.1% vs. 17.8%, *p* = 0.006), or distant metastasis at the time of diagnosis (29% vs. 15.3%, *p* = 0.032) (Table 1).

### 3.2. Histopathological Assessment and BRAF Mutation Analysis

**Figure 1:**
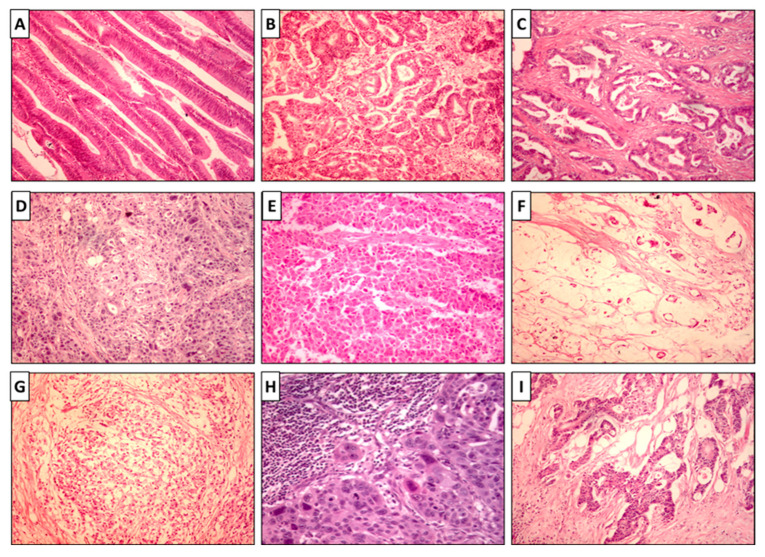
Histopathological findings in colon carcinoma. (**A**) Colonic adenoma with a moderate to high degree of dysplasia (×100). (**B**) Well-differentiated colon adenocarcinoma formed of more than 95% of invasive irregular separate glands (×200). (**C**) Moderately differentiated colonic adenocarcinoma (Grade 2) showing irregular fused glands infiltrating the wall (×200). (**D**) Poorly differentiated colonic adenocarcinoma (Grade 3) showing diffuse sheets of pleomorphic anaplastic cells with few irregular acinar-like structures (×200). (**E**) Poorly differentiated colonic adenocarcinoma (Grade 3) formed of sheets of undifferentiated cells with no evidence of acinar formations (×200). (**F**) Mucinous colonic carcinoma showed lakes and pools with mucin with floating malignant cells and fragments of acini (×200). (**G**) Signet ring carcinoma of the colon formed of malignant cells with signet ring appearance (×200). (**H**) Mesenteric lymph node containing metastatic deposits of colonic carcinoma (×200). (**I**) Deep invasion of colonic carcinoma in the colonic wall down to subserosal fat (×100).

**Figure 2:**
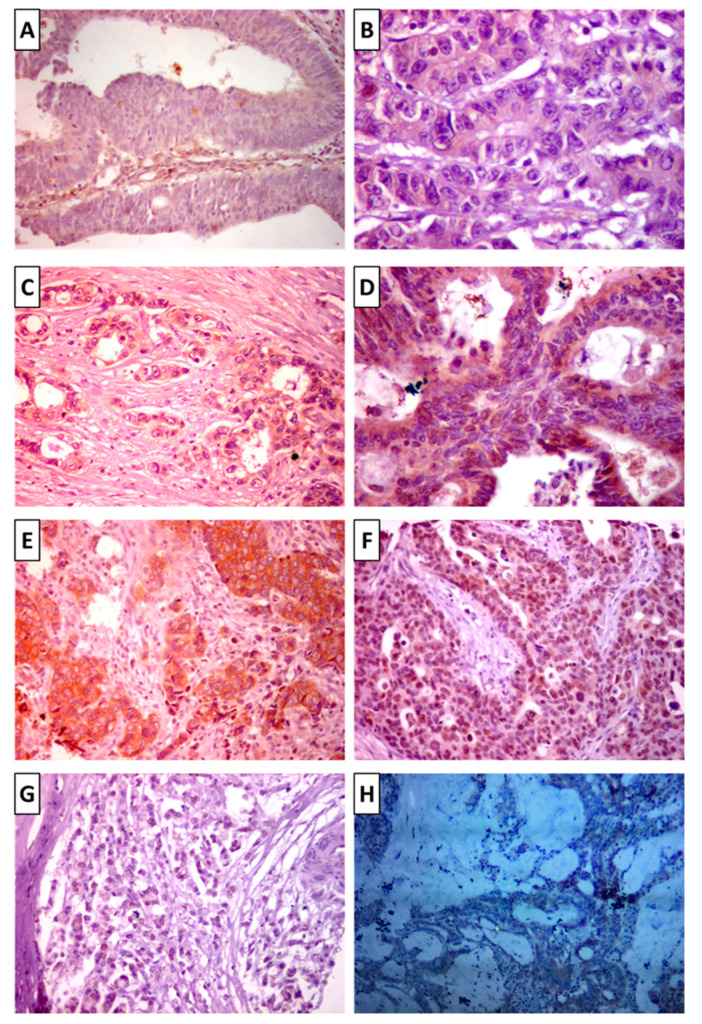
Immunohistochemistry staining for Braf mutant (BRAF^V600E^) protein. (**A**) Well-differentiated adenocarcinoma showing negative staining of BRAF (×200). (**B**) Well-differentiated adenocarcinoma showing weak cytoplasmic staining (×400). (**C**) Moderately differentiated adenocarcinoma showing moderate cytoplasmic staining (×200). (**D**) Moderately differentiated adenocarcinoma showing strong cytoplasmic staining (×400). (**E**) Poorly differentiated adenocarcinoma showing cytoplasmic staining (×200). (**F**) Poorly differentiated adenocarcinoma showing nuclear staining (×200). (**G**) Signet ring nuclei showing scattered few positively stained nuclei (200). (**H**) Mucinous adenocarcinoma showing negative staining of the BRAF mutation (×100).

Representative examples of the slide examination using hematoxylin and eosin are depicted in Figure 1. Immunohistochemistry analysis of 351 colon cancer tissue specimens for mutant BRAF revealed positive staining in 60 samples, while 291 samples were not stained (Figure 2). The expression was inversely related to the degree of differentiation (greater expression with poorly and undifferentiated tumors). The staining was cytoplasmic with/or without nuclear staining. Mucinous adenocarcinoma showed negative staining, while the signet ring showed scattered positivity.

### 3.3. Genotype and Allele Frequencies of the *LINC-ROR* Variant in CRC Patients

**Figure 3:**
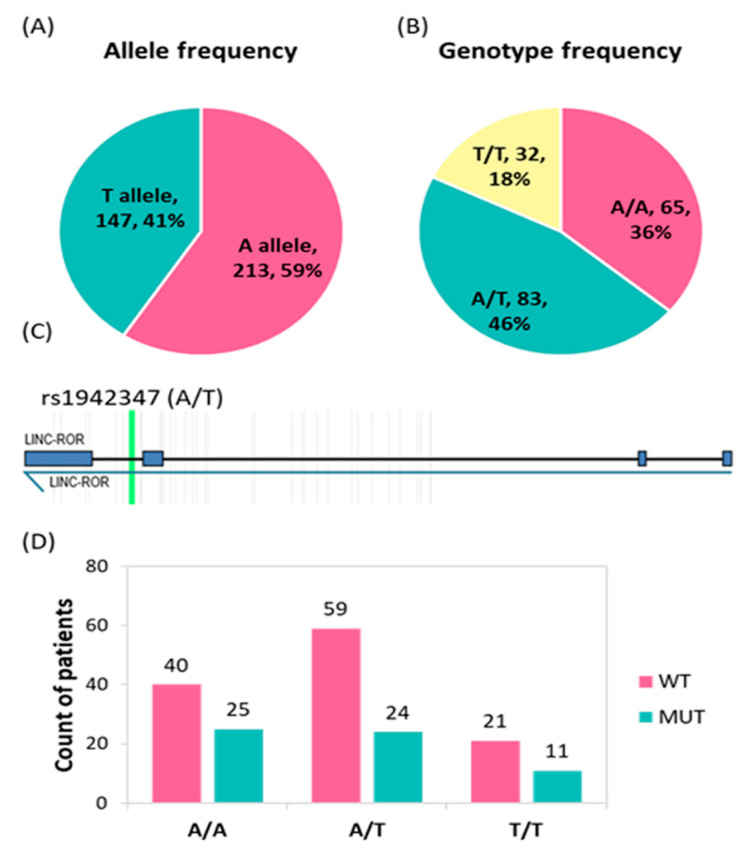
Genotype and allele frequencies of the *LINC-ROR* rs1942347 variant. Data are presented as a frequency and percentage. A two sided-Chi-square test was used. Statistical analysis was set at a *p*-value below 0.05. (**A**) Allele frequency of patients with CC, (**B**) Genotype frequency of 180 patients with CC. (**C**) Gene variant location within the *LINC_ROR* intron. (**D**) Relationship between the BRAF mutation and genotype results (*p* = 0.46).

The genotype frequency of rs1942347 was in accordance with HWE (*p* = 0.54). MAF (T allele) accounted for 0.41 in the study population (Figure 3A). According to the 1000 Genome Project, the same allele frequencies were 0.42 in East Asians, 0.23 in South Asians, 0.35 in Americans, 0.31 in Europeans, and 0.86 in Africans. Genotype frequencies for T/T, A/T, and A/A were 18% (N = 32), 46% (N = 83), and 36% (N = 65), respectively (Figure 3B). The intronic study variant is located at chromosome 18:57057227 according to the (GRCh38.p13) build (Figure 3C). Testing the association of different genotypes of the studied variant with the BRAF mutation status revealed insignificant results (Figure 3D).

### 3.4. Prognostic Value of the *LINC-ROR* Genotypes in CRC

The *LINC-ROR* rs1942347*A variant was associated with an earlier onset of colon cancer in a dose-dependent manner (*p* = 0.039). Patients with A/A and A/T were more prevalent (61.5% and 49.4%) compared to T/T carriers (34.4%) (Table 2). There was no significant difference regarding patient’s sex (*p* = 0.18), tumor site (*p* = 0.52), histopathological diagnosis (*p* = 0.60), or BRAF status (*p* = 0.46). However, rs1942347*A polymorphism was associated with a high pathological grade (A/A: 30.8%, A/T: 27.7%, T/T: 3.1%, *p* = 0.008), larger tumor size (A/A: 36.9%, A/T: 25.3%, T/T: 9.4%, *p* = 0.014), distant metastasis (A/A: 29.2%, A/T: 18.1%, T/T: 6.3%, *p* = 0.024), and mortality (A/A: 47.7%, A/T: 31.3%, T/T: 15.6%, *p* = 0.005) (Table 2). In this sense, the study variant was significantly associated with poor prognostic indices in the enrolled cases of colon cancer.

As shown in Table 3, multiple logistic regression analysis adjusted by sex and BRAF mutation showed that A variant carriers had a 3 times greater risk of early onset of cancer under the homozygote comparison model (A/A vs. T/T: OR = 3.13, 95%CI = 1.28–7.69, *p* = 0.034).

### 3.5. Survival Analysis

Overall analysis showed that cancer patient carriers of rs1942347*A variant had a higher risk of mortality under the heterozygote comparison (A/T vs. T/T: OR = 2.13, 95%CI = 1.08–4.35, *p* = 0.003), homozygote comparison (A/A vs. T/T: OR = 5.0, 95%CI = 1.69–14.29, *p* =0.003), dominant model (A/T-A/A vs. T/T: OR = 3.33, 95%CI = 1.20–9.09, *p* = 0.003), and recessive model (A/A vs. T/T-A/T: OR = 2.63, 95%CI = 1.37–5.0, *p* = 0.011) (Table 4). According to the AIC value, the codominant comparison was the best model. Stratified analysis by BRAF status revealed that the presence of the ancestor allele of the *LINC-ROR* rs1942347 variant conferred protection in BRAF mutant CC patients and was associated with a 73–93% reduced risk of mortality under the heterozygote comparison (OR = 0.27, 95%CI = 0.08–0.90) and homozygote comparison models (OR = 0.07, 95%CI = 0.01–0.68). In contrast, the same SNP did not show an association with survival in the BRAF negative group (Supplementary Table S3).

**Figure 4:**
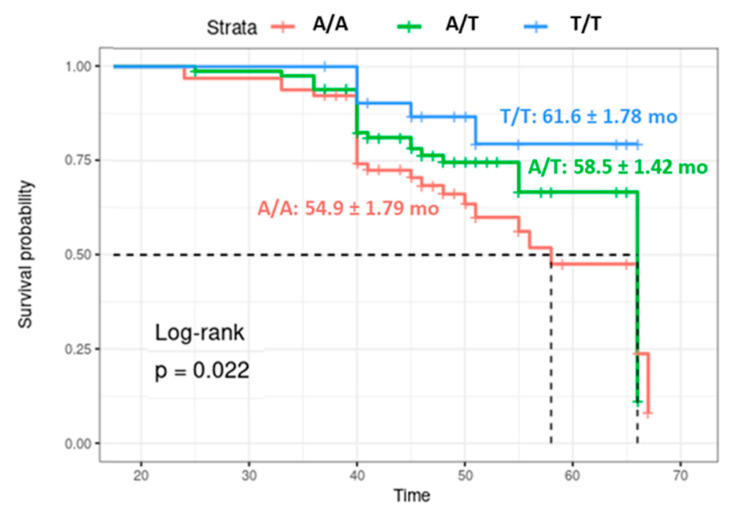
Kaplan–Meier survival curves for *LINC-ROR* genotypes. The *X*-axis represents the overall survival in months. The Log-Rank test was used for overall and pairwise comparison. The Bonferroni test was applied for *p*-value adjustment. Dashed black lines represent the median times.

**Figure 5:**
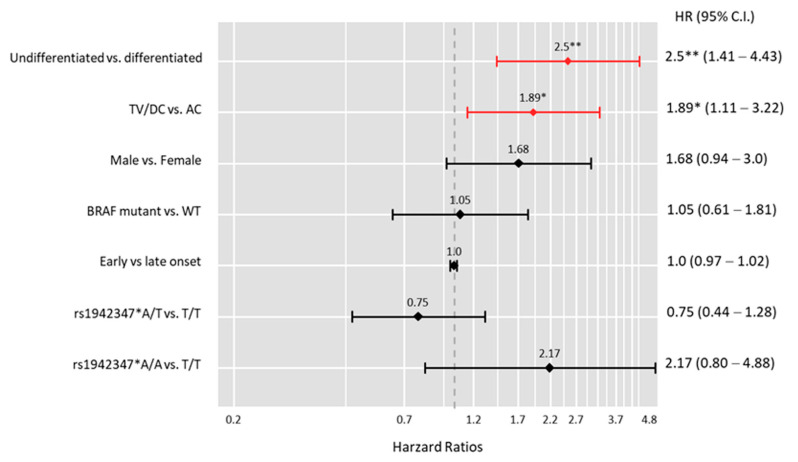
Multivariate Cox regression analysis for overall survival. * *p* = 0.018, ** *p* = 0.001. TV: transverse colon; DC: descending colon; AC: ascending colon; WT: wild type. Hazards ratios (HRs) and 95% confidence intervals (CIs) are reported.

Kaplan–Meier curves represent the survival times for each genotype (Figure 4). Carriers of the A/A genotype (54.9 ± 1.79 months) had shorter survival times than T/T cohorts (61.6 ± 1.78 months, *p* = 0.022). The univariate Cox regression model revealed that A/A was associated with a 3.5 times greater mortality risk than T/T (HR = 3.57, 95%CI = 1.35–9.09, *p* = 0.010). However, after adjustment by multiple Cox regression analysis, the risk was not significant (HR = 2.17, 95%CI = 0.80–4.88) (Figure 5).

## 4. Discussion

As a recently identified molecular target for cancer therapy, lncRNAs provide an outstanding opportunity to impact several aspects of cancer progression, including colon cancer [32]. Our study, for the first time, reports the association of the *LINC-ROR* rs1942347 variant with poor colon cancer outcomes. More specifically, the rs1942347*A variant carriers showed a three times greater risk for earlier onset colon cancer than counterpart allele carriers. Moreover, the rs1942347*A polymorphism was associated with larger tumor size, high pathological grade, distant metastasis, and mortality.

Several association studies have confirmed the role of the lncRNAs variants in multiple cancers, including CC [33,34,35]. For example, the *H19* rs2839698*A allele was significantly associated with an increased risk of CC in Chinese by modifying the folding structure and targeted microRNAs of H19 [36]. Similarly, the lncRNA colorectal cancer-associated transcript 1 (*CCAT1*) rs67085638C/T and rs7013433A/T variants were found to be associated with increased CC risk and advanced stage, respectively, in the same population [37]. The *lnc-LAMC2-1:1* rs2147578 polymorphism was found to be a genetic modifier for CC development via changing the sponging effect of this lncRNA on miR-128-3p [38]. Furthermore, the lncRNA *PCAT1* rs2632159 variant was reported to influence CRC risk by altering the binding of the transcriptional factors: EBF, LUN-1, and TCF12, thereby upregulating PCAT1 in the tissues and potentiating its oncogenic role [33]. The metastasis-associated lung adenocarcinoma transcript 1 (*MALAT1*) rs664589G allele was associated with gene upregulation and accelerated colon cancer growth/metastasis [34]. In contrast, the lncRNA HOXA transcript at the distal tip (*HOTTIP*) rs17501292 variant showed improvement in overall survival of cancer patients with ulcerative or invasive tumors [39]. Collectively, these studies, among others, support the potential utility of lncRNA SNPs as genetic biomarkers for CC risk and progression.

The lncRNA variants may impact gene expression and RNA processing, and/or modulate the secondary structure that influences the interacting molecular network and downstream targets [40,41], culminating in cancer development and progression [5,42]. In an attempt to predict the impact of the intronic *LINC-ROR* rs1942347A/T variant on cancer outcome, we ran the HaploReg v4.1; (https://pubs.broadinstitute.org/mammals/haploreg/haploreg.php) (last accessed 5 March 2022), a validated online bioinformatics tool specified for exploring annotations of the non-coding genome variants based on the 1000 Genomes Project, and on expression quantitative trait locus studies [43]. We found that rs1942347 was in strong linkage disequilibrium (r^2^ ≥ 8) with the other 12 SNPs on the same *LINC-ROR* gene locus on chromosome 18 (Supplementary Table S4). This suggests that the potential effect of this variant on the overall cancer phenotype could be due to complex interactions with other polymorphisms, which is more impactful than the independent main effects of one SNP [33]. Interestingly, one of the linked SNPs, rs2027701, has been associated with worse concurrent chemoradiotherapy efficacy at the lymph node of patients with nasopharyngeal carcinoma [44]. Moreover, the analysis revealed that this variant might be associated with 10 altered DNA motifs, which could partly explain the significant impact and association of this variant with poor prognostic indices identified in the current study. Further in vitro functional studies are recommended to confirm these findings.

After stratifying our patient cohort by BRAF status, we found that the presence of the ancestor allele (T) of the *LINC-ROR* rs1942347 variant conferred protection in BRAF mutant CC patients and was associated with a 73–93% reduced risk of mortality. Interestingly, Wang et al. demonstrated “the p53 signaling pathway as the most highly enriched pathway among the BRAF mutation-related genes” [45]. As Zhang et al. reported evidence that the LINC-RoR is a potent negative regulator of p53 translation through direct interaction with the heterogeneous nuclear ribonucleoprotein I (hnRNP I) with subsequent inhibition of p53-mediated cell cycle arrest and apoptosis [46], it can be speculated that in the case of carriers of the ancestral allele (non-pathological one) that is not associated with dysregulated ROR expression, the p53 regulatory mechanisms on the cell apoptosis will be issued, improving the cancer cell outcome.

Although the present study was the first to report a significant association between the *LINC-ROR* rs1942347 variant and poor prognosis of patients with colon cancer, it lacks the functional and mechanistic works that unravel the specific role of the studied variant on *LINC-ROR* gene expression and/or the impacted downstream targets in colon cancer, which is planned for future work. Furthermore, the reproducibility of the findings should be confirmed in other ethnic populations.

## 5. Conclusions

This study unraveled the association of the lncRNA-ROR rs1942347A/T variant with poor prognosis in terms of high pathological grade, larger tumor size, distant metastasis, and mortality in patients with colon cancer. Further studies are required to explore the influence of this variant on gene expression and to study its potential association with chemoresistance in this type of cancer.

## Figures and Tables

**Table 1:** Baseline characteristics of the study population according to survival.

Variable		Total (n = 180)	Survived (n = 118)	Died (n = 62)	*p*-Value
Age (y)	≤60	92 (51.1)	57 (48.3)	35 (56.5)	0.34
	>60	88 (48.9)	61 (51.7)	27 (43.5)	
Sex	Male	111 (61.7)	68 (57.6)	43 (69.4)	0.14
	Female	69 (38.3)	50 (42.4)	19 (30.6)	
Location	Right	97 (53.9)	73 (61.9)	24 (38.7)	**0.004**
	Transverse/left	83 (46.1)	45 (38.1)	38 (61.3)	
Type	Adenocarcinoma	128 (71.1)	82 (69.5)	46 (74.2)	0.60
	Others	52 (28.9)	36 (30.5)	16 (25.8)	
Grade	G1	136 (75.6)	97 (82.2)	39 (62.9)	**0.006**
	G2/G3	44 (24.4)	21 (17.8)	23 (37.1)	
T stage	T1/2	132 (73.3)	87 (73.7)	45 (72.6)	0.86
	T3/4	48 (26.7)	31 (26.3)	17 (27.4)	
N stage	Negative	72 (40)	42 (35.6)	30 (48.4)	0.11
	Positive	108 (60)	76 (64.4)	32 (51.6)	
M stage	Negative	144 (80)	100 (84.7)	44 (71)	**0.032**
	Positive	36 (20)	18 (15.3)	18 (29)	
Duke’s stage	A/B	111 (61.7)	73 (61.9)	38 (61.3)	1.00
	C/D	69 (38.3)	45 (38.1)	24 (38.7)	
BRAF mutation	Wild type	120 (66.7)	80 (67.8)	40 (64.5)	0.74
	Mutant	60 (33.3)	38 (32.2)	22 (35.5)	
Relapse	No	127 (70.6)	89 (75.4)	38 (61.3)	0.06
	Yes	53 (29.4)	29 (24.6)	24 (38.7)	

Data are presented as frequencies (percentages). A two sided-Chi-square test was used. Bold values indicate statistical significance at a *p*-value < 0.05.

**Table 2:** Association of *LINC-ROR* rs1942347 genotypes with clinical and pathological features.

Variable		Number	T/T (N = 32)	A/T (N = 83)	A/A (N = 65)	*p*-Value
Age (y)	≤60	92	11 (34.4)	41 (49.4)	40 (61.5)	**0.039**
	>60	88	21 (65.6)	42 (50.6)	25 (38.5)	
Sex	Female	111	17 (53.1)	57 (68.7)	37 (56.9)	0.18
	Male	69	15 (46.9)	26 (31.3)	28 (43.1)	
Location	Right	97	19 (59.4)	41 (49.4)	37 (56.9)	0.52
	Transverse/left	83	13 (40.6)	42 (50.6)	28 (43.1)	
Type	Adenocarcinoma	128	25 (78.1)	57 (68.7)	46 (70.8)	0.60
	Others	52	7 (21.9)	26 (31.3)	19 (29.2)	
Grade	G1	136	31 (96.9)	60 (72.3)	45 (69.2)	**0.008**
	G2/3	44	1 (3.1)	23 (27.7)	20 (30.8)	
T stage	T1/2	132	29 (90.6)	62 (74.7)	41 (63.1)	**0.014**
	T3/4	48	3 (9.4)	21 (25.3)	24 (36.9)	
Lymph node metastasis	Negative	72	12 (37.5)	34 (41)	26 (40)	0.94
	Positive	108	20 (62.5)	49 (59)	39 (60)	
Distant metastasis	Negative	144	30 (93.8)	68 (81.9)	46 (70.8)	**0.024**
	Positive	36	2 (6.3)	15 (18.1)	19 (29.2)	
Duke’s stage	A/B	111	25 (78.1)	51 (61.4)	35 (53.8)	0.06
	C/D	69	7 (21.9)	32 (38.6)	30 (46.2)	
BRAF mutation	Wild type	120	21 (65.6)	59 (71.1)	40 (61.5)	0.46
	Mutant	60	11 (34.4)	24 (28.9)	25 (38.5)	
Relapse	Negative	127	22 (68.8)	62 (74.7)	43 (66.2)	0.51
	Positive	53	10 (31.3)	21 (25.3)	22 (33.8)	
Mortality	Negative	118	27 (84.4)	57 (68.7)	34 (52.3)	**0.005**
	Positive	62	5 (15.6)	26 (31.3)	31 (47.7)	
DFS (months)	Prolonged (≥48)	61	15 (46.9)	28 (33.7)	18 (27.7)	0.17
	Short (<48)	119	17 (53.1)	55 (66.3)	47 (72.3)	
OS (months)	Prolonged (≥48)	87	19 (59.4)	40 (48.2)	28 (43.1)	0.31
	Short (<48)	93	13 (40.6)	43 (51.8)	37 (56.9)	

Data are presented as a frequency (percentage). A two sided-Chi-square test was used. Bold values indicate a statistically significant *p*-value below 0.05. DFS: disease-free survival; OS: overall survival.

**Table 3:** Genetic inheritance association models for the *LINC-ROR* gene variant and early cancer risk.

Frequency	Genotype	Late Onset	Early Onset	OR (95%CI)	*p*-Value	AIC
Codominant	T/T	21 (23.9%)	11 (12%)	*Reference*	**0.034**	252.1
	A/T	42 (47.7%)	41 (44.6%)	1.64 (0.84–3.23)		
	A/A	25 (28.4%)	40 (43.5%)	**3.13 (1.28–7.69)**		
Dominant	T/T	21 (23.9%)	11 (12%)	*Reference*	**0.031**	252.2
	A/A-A/T	67 (76.1%)	81 (88%)	**2.38 (1.06–5.26)**		
Recessive	A/T-T/T	63 (71.6%)	52 (56.5%)	*Reference*	**0.033**	252.3
	A/A	25 (28.4%)	40 (43.5%)	**1.96 (1.05–3.7)**		

Multivariate regression analysis was applied. The adjusted odds ratio (OR) and 95% confidence interval (CI) are shown. The models were adjusted for age, sex, and BRAF mutation. AIC: Akaike information criterion; used for evaluating how well a model fits the data it was generated from it. AIC was calculated from the number of independent variables used to build the model. Bold values indicate a statistically significant *p*-value below 0.05.

**Table 4:** Genetic inheritance association models for the *LINC-ROR* gene variant and mortality risk.

Frequency	Genotype	Survived	Died	OR (95%CI)	*p*-Value	AIC
Codominant	T/T	27 (22.9%)	5 (8.1%)	*Reference*	**0.0037**	230.0
	A/T	57 (48.3%)	26 (41.9%)	**2.13 (1.08–4.35)**		
	A/A	34 (28.8%)	31 (50%)	**5.0 (1.69–14.29)**		
Dominant	T/T	27 (22.9%)	5 (8.1%)	*Reference*	**0.011**	232.7
	A/A-A/T	91 (77.1%)	57 (91.9%)	**3.33 (1.2–9.09)**		
Recessive	A/T-T/T	84 (71.2%)	31 (50%)	*Reference*	**0.0035**	230.6
	A/A	34 (28.8%)	31 (50%)	**2.63 (1.37–5)**		

Multivariate regression analysis was applied. The adjusted odds ratio (OR) and 95% confidence interval (CI) are shown. The models were adjusted for age, sex, and BRAF mutation. AIC: Akaike information criterion; used for evaluating how well a model fits the data it was generated from it. AIC was calculated from the number of independent variables used to build the model. Bold values indicate a statistically significant *p*-value below 0.05.

## Data Availability

All generated data in this study are included in the article and Supplementary Material.

## Supplementary Materials

The following supporting information can be downloaded at: https://www.mdpi.com/article/10.3390/biom12040569/s1, Table S1: Characteristics of the *LINC-ROR*-related intronic variants cited in previous literature; Table S2: Characteristics of propensity-matched cohorts; Table S3: Codominant association model for *LINC-ROR* gene variant and mortality risk stratified by BRAF mutation; Table S4: Impact and linkage disequilibrium (LD) of *LINC-ROR* rs1942347 variant on chromosome 18 with other variants (r2 ≥ 0.8) on the same chromosome.

## References

[1] Rawla P., Sunkara T., Barsouk A. (2019). Epidemiology of colorectal cancer: Incidence, mortality, survival, and risk factors. Prz. Gastroenterol..

[2] Siegel R.L., Miller K.D., Goding Sauer A., Fedewa S.A., Butterly L.F., Anderson J.C., Cercek A., Smith R.A., Jemal A. (2020). Colorectal cancer statistics, 2020. CA Cancer J. Clin..

[3] Xi Y., Xu P. (2021). Global colorectal cancer burden in 2020 and projections to 2040. Transl. Oncol..

[4] Kormi S.M.A., Ardehkhani S., Kerachian M.A. (2017). New insights into colorectal cancer screening and early detection tests. Colorectal. Cancer.

[5] Poursheikhani A., Abbaszadegan M.R., Kerachian M.A. (2021). Mechanisms of long non-coding RNA function in colorectal cancer tumorigenesis. Asia Pac. J. Clin. Oncol..

[6] Diori Karidio I., Sanlier S.H. (2021). Reviewing cancer’s biology: An eclectic approach. J. Egypt. Natl. Cancer Inst..

[7] Ibrahiem A.T., Fawzy M.S., Abu AlSel B.T., Toraih E.A. (2021). Prognostic value of BRAF/MIR-17 signature and B-Raf protein expression in patients with colorectal cancer: A pilot study. J. Clin. Lab. Anal..

[8] Wang R., Du L., Yang X., Jiang X., Duan W., Yan S., Xie Y., Zhu Y., Wang Q., Wang L. (2016). Identification of long non-coding RNAs as potential novel diagnosis and prognosis biomarkers in colorectal cancer. J. Cancer Res. Clin. Oncol..

[9] Liao Z., Nie H., Wang Y., Luo J., Zhou J., Ou C. (2021). The Emerging Landscape of Long Non-Coding RNAs in Colorectal Cancer Metastasis. Front. Oncol..

[10] Thiele J.A., Hosek P., Kralovcova E., Ostasov P., Liska V., Bruha J., Vycital O., Rosendorf J., Opattova A., Horak J. (2018). lncRNAs in Non-Malignant Tissue Have Prognostic Value in Colorectal Cancer. Int. J. Mol. Sci..

[11] Toraih E.A., El-Wazir A., Hussein M.H., Khashana M.S., Matter A., Fawzy M.S., Hosny S. (2019). Expression of long intergenic non-coding RNA, regulator of reprogramming, and its prognostic value in patients with glioblastoma. Int. J. Biol. Mark..

[12] Chen W., Yang J., Fang H., Li L., Sun J. (2020). Relevance Function of Linc-ROR in the Pathogenesis of Cancer. Front. Cell Dev. Biol..

[13] Toraih E.A., El-Wazir A., Ageeli E.A., Hussein M.H., Eltoukhy M.M., Killackey M.T., Kandil E., Fawzy M.S. (2020). Unleash multifunctional role of long non-coding RNAs biomarker panel in breast cancer: A predictor classification model. Epigenomics.

[14] Li X., Chen W., Jia J., You Z., Hu C., Zhuang Y., Lin Z., Liu Y., Yang C., Xu R. (2020). The Long Non-Coding RNA-RoR Promotes the Tumorigenesis of Human Colorectal Cancer by Targeting miR-6833-3p Through SMC4. Onco Targets Ther..

[15] Fawzy M.S., Toraih E.A., El-Wazir A., Hosny M.M., Badran D.I., El Kelish A. (2021). Long intergenic non-coding RNA, regulator of reprogramming (*LINC-ROR*) over-expression predicts poor prognosis in renal cell carcinoma. Arch. Med. Sci..

[16] Ma Y.L., Wang C.Y., Guan Y.J., Gao F.M. (2020). Long non-coding RNA ROR promotes proliferation and invasion of colorectal cancer by inhibiting tumor suppressor gene NF2 through interacting with miR-223-3p. Eur. Rev. Med. Pharmacol. Sci..

[17] Yang P., Yang Y., An W., Xu J., Zhang G., Jie J., Zhang Q. (2017). The long non-coding RNA-ROR promotes the resistance of radiotherapy for human colorectal cancer cells by targeting the p53/miR-145 pathway. J. Gastroenterol. Hepatol..

[18] Lv Z., Xu Q., Yuan Y. (2017). A systematic review and meta-analysis of the association between long non-coding RNA polymorphisms and cancer risk. Mutat. Res. Rev. Mutat. Res..

[19] Luo C., Cao J., Peng R., Guo Q., Ye H., Wang P., Wang K., Song C. (2018). Functional Variants in Linc-ROR are Associated with mRNA Expression of Linc-ROR and Breast Cancer Susceptibility. Sci. Rep..

[20] Holmans P.A., Riley B., Pulver A.E., Owen M.J., Wildenauer D.B., Gejman P.V., Mowry B.J., Laurent C., Kendler K.S., Nestadt G. (2009). Genomewide linkage scan of schizophrenia in a large multicenter pedigree sample using single nucleotide polymorphisms. Mol. Psychiatry.

[21] Amin M.B., Greene F.L., Edge S.B., Compton C.C., Gershenwald J.E., Brookland R.K., Meyer L., Gress D.M., Byrd D.R., Winchester D.P. (2017). The Eighth Edition AJCC Cancer Staging Manual: Continuing to build a bridge from a population-based to a more “personalized” approach to cancer staging. CA Cancer J. Clin..

[22] Aust D.E. (2011). WHO classification 2010 for the lower gastrointestinal tract: What is new?. Pathologe.

[23] Akkoca A.N., Yanık S., Ozdemir Z.T., Cihan F.G., Sayar S., Cincin T.G., Cam A., Ozer C. (2014). TNM and Modified Dukes staging along with the demographic characteristics of patients with colorectal carcinoma. Int. J. Clin. Exp. Med..

[24] Choden S., Keelawat S., Jung C.K., Bychkov A. (2020). VE1 Immunohistochemistry Improves the Limit of Genotyping for Detecting. Cancers.

[25] Rashid F.A., Tabassum S., Khan M.S., Ansari H.R., Asif M., Sheikh A.K., Sameer Aga S. (2021). VE1 immunohistochemistry is an adjunct tool for detection of BRAF. J. Clin. Lab. Anal..

[26] Dvorak K., Higgins A., Palting J., Cohen M., Brunhoeber P. (2019). Immunohistochemistry with Anti-BRAF V600E (VE1) Mouse Monoclonal Antibody is a Sensitive Method for Detection of the BRAF V600E Mutation in Colon Cancer: Evaluation of 120 Cases with and without KRAS Mutation and Literature Review. Pathol. Oncol. Res..

[27] Coordinators N.R. (2016). Database resources of the National Center for Biotechnology Information. Nucleic Acids Res..

[28] Mohammad H.M.F., Abdelghany A.A., Al Ageeli E., Kattan S.W., Hassan R., Toraih E.A., Fawzy M.S., Mokhtar N. (2021). Long Non-Coding RNAs Gene Variants as Molecular Markers for Diabetic Retinopathy Risk and Response to Anti-VEGF Therapy. Pharmgenom. Pers. Med..

[29] Elwazir M.Y., Hussein M.H., Toraih E.A., Al Ageeli E., Esmaeel S.E., Fawzy M.S., Faisal S. (2022). Association of Angio-LncRNAs MIAT rs1061540/MALAT1 rs3200401 Molecular Variants with Gensini Score in Coronary Artery Disease Patients Undergoing Angiography. Biomolecules.

[30] Solé X., Guinó E., Valls J., Iniesta R., Moreno V. (2006). SNPStats: A web tool for the analysis of association studies. Bioinformatics.

[31] MS F., MH H., EZ A., HA Y., HM I., EA T. (2016). Association of MicroRNA-196a2 Variant with Response to Short-Acting β2-Agonist in COPD: An Egyptian Pilot Study. PLoS ONE.

[32] Arun G., Diermeier S.D., Spector D.L. (2018). Therapeutic Targeting of Long Non-Coding RNAs in Cancer. Trends Mol. Med..

[33] Yang M.L., Huang Z., Wu L.N., Wu R., Ding H.X., Wang B.G. (2019). lncRNA-PCAT1 rs2632159 polymorphism could be a biomarker for colorectal cancer susceptibility. Biosci. Rep..

[34] Wu S., Sun H., Wang Y., Yang X., Meng Q., Yang H., Zhu H., Tang W., Li X., Aschner M. (2019). MALAT1 rs664589 Polymorphism Inhibits Binding to miR-194-5p, Contributing to Colorectal Cancer Risk, Growth, and Metastasis. Cancer Res..

[35] Wang Y., Wu S., Yang X., Li X., Chen R. (2019). Association between polymorphism in the promoter region of lncRNA GAS5 and the risk of colorectal cancer. Biosci. Rep..

[36] Li S., Hua Y., Jin J., Wang H., Du M., Zhu L., Chu H., Zhang Z., Wang M. (2016). Association of genetic variants in lncRNA H19 with risk of colorectal cancer in a Chinese population. Oncotarget.

[37] Li Y., Jing F., Ding Y., He Q., Zhong Y., Fan C. (2018). Long non-coding RNA CCAT1 polymorphisms are associated with the risk of colorectal cancer. Cancer Genet..

[38] Gong J., Tian J., Lou J., Ke J., Li L., Li J., Yang Y., Gong Y., Zhu Y., Zhang Y. (2016). A functional polymorphism in lnc-LAMC2-1:1 confers risk of colorectal cancer by affecting miRNA binding. Carcinogenesis.

[39] Lv Z., Xu Q., Sun L., Wen J., Fang X., Xing C., Yuan Y. (2019). Four novel polymorphisms in long non-coding RNA HOTTIP are associated with the risk and prognosis of colorectal cancer. Biosci. Rep..

[40] Cheetham S.W., Gruhl F., Mattick J.S., Dinger M.E. (2013). Long non-coding RNAs and the genetics of cancer. Br. J. Cancer.

[41] Ling H., Vincent K., Pichler M., Fodde R., Berindan-Neagoe I., Slack F.J., Calin G.A. (2015). Junk DNA and the long non-coding RNA twist in cancer genetics. Oncogene.

[42] Jiang D., Jin M., Ye D., Li Y., Jing F., Zhang X., Li Q., Chen K. (2020). Polymorphisms of a novel long non-coding RNA RP11-108K3.2 with colorectal cancer susceptibility and their effects on its expression. Int. J. Biol. Mark..

[43] Ward L.D., Kellis M. (2012). HaploReg: A resource for exploring chromatin states, conservation, and regulatory motif alterations within sets of genetically linked variants. Nucleic Acids Res..

[44] Wang Y., Guo Z., Zhao Y., Jin Y., An L., Wu B., Liu Z., Chen X., Zhou H., Wang H. (2017). Genetic polymorphisms of lncRNA-p53 regulatory network genes are associated with concurrent chemoradiotherapy toxicities and efficacy in nasopharyngeal carcinoma patients. Sci. Rep..

[45] Wang Q., Yang H., Wu L., Yao J., Meng X., Jiang H., Xiao C., Wu F. (2016). Identification of Specific Long Non-Coding RNA Expression: Profile and Analysis of Association with Clinicopathologic Characteristics and BRAF Mutation in Papillary Thyroid Cancer. Thyroid.

[46] Zhang A., Zhou N., Huang J., Liu Q., Fukuda K., Ma D., Lu Z., Bai C., Watabe K., Mo Y.Y. (2013). The human long non-coding RNA-RoR is a p53 repressor in response to DNA damage. Cell Res..

