# Supplementary material for: Prognostic Value of LINC-ROR (rs1942347) Variant in Patients with Colon Cancer Harboring BRAF Mutation: A Propensity Score-Matched Analysis
Source: Biomolecules. 2022 Apr 12;12(4):569. doi: 10.3390/biom12040569 (PMC9028515; doi:10.3390/biom12040569)
Supplement: Supplementary file 1 [file biomolecules-12-00569-s001.zip › biomolecules-1664183-supplementary.pdf]

Table S1. Characteristics of the LINC-ROR-related intronic variants cited in previous literature

SNP ID	Alleles	Type	MAF	Genomic location	Overlapping	Disease	PMID
rs6420545	T/C	Intronic	0.34	18:57057008	3 transcripts	Breast cancer	29549263
**rs1942347**	A/T	Intronic	0.47	18:57057227	3 transcripts	NA	NA
rs1942348	T/C	Intronic	0.44	18:57057466	3 transcripts	Breast cancer	29549263
rs732982	G/A	Intronic	0.18	18:57060228	7 transcripts	Schizophrenia	19223858
rs4801078	T/C	Intronic	0.26	18:57061452	7 transcripts	Breast cancer	29549263
rs9636089	T/C	Intronic	0.32	18:57062094	7 transcripts	Breast cancer	29549263

The current studied variant is bold. SNP: Single nucleotide polymorphism; MAF: Minor allele frequency; PMID: PubMed ID; NA: Non-applicable.

Table S2. Characteristics of propensity-matched cohorts

Variable		Wild type BRAF (n = 120)	Mutant BRAF (n = 60)	*p*-value
**Age (y)**	**≤60**	63 (52.5)	29 (48.3)	0.63
	**>60**	57 (47.5)	31 (51.7)	
**Sex**	**Male**	76 (63.3)	35 (58.3)	0.52
	**Female**	44 (36.7)	25 (41.7)	
**Location**	**Right**	66 (55)	31 (51.7)	0.75
	**Transverse/left**	54 (45)	29 (48.3)	
**Type**	**Adenocarcinoma**	89 (74.2)	39 (65)	0.22
	**Others**	31 (25.8)	21 (35)	
**Grade**	**G1**	95 (79.2)	41 (68.3)	0.14
	**G2/G3**	25 (20.8)	19 (31.7)	
**T stage**	**T1/2**	93 (77.5)	39 (65)	0.11
	**T3/4**	27 (22.5)	21 (35)	
**N stage**	**Negative**	50 (41.7)	22 (36.7)	0.62
	**Positive**	70 (58.3)	38 (63.3)	
**M stage**	**Negative**	96 (80)	48 (80)	1.0
	**Positive**	24 (20)	12 (20)	
**Duke's stage**	**A/B**	78 (65)	33 (55)	0.19
	**C/D**	42 (35)	27 (45)	

Data are presented as frequencies (percentages). A two sided-Chi-square test was used. Statistical analysis was set at a *p*-value below 0.05.

Supplementary Table S3. Codominant association model for *LINC-ROR* gene variant and mortality risk stratified by BRAF mutation status

Genotype	Wild-type BRAF			Mutant BRAF		
	**Survived**	**Died**	**OR (95%CI)**	**Survived**	**Died**	**OR (95%CI)**
A/A	23	17	*Reference*	11	14	*Reference*
A/T	40	19	0.63 (0.27-1.45)	17	7	**0.27 (0.08-0.90)**
T/T	17	4	0.32 (0.09-1.12)	10	1	**0.07 (0.01-0.68)**

Multivariate regression analysis was applied. Adjusted odds ratio (OR) and 95% confidence interval (CI) are shown. The models were adjusted for age and sex.

Table S4. Impact and linkage disequilibrium (LD) of *LINC-ROR* rs1942347 variant on chromosome 18 with other variants ($r^2 \geq 0.8$) on the same chromosome

pos (hg38)	LD (r^2)	LD (D')	variant	Ref	Alt	AFR freq.	AMR freq.	ASN freq.	EUR freq.	Proteins bound	Motifs changed	GENCODE genes
57052460	0.97	0.99	rs8093490	A	G	0.85	0.34	0.4	0.3	CTCF, RAD21, SMC3	GCM, Gcm1	2.1kb 3' of LINC-ROR
57052965	0.85	0.99	rs9953564	G	A	0.85	0.3	0.34	0.28			1.6kb 3' of LINC-ROR
57053146	0.85	0.99	rs9945715	T	C	0.85	0.31	0.34	0.28		Rad21, SETDB1, Znf143	1.4kb 3' of LINC-ROR
57053429	0.85	0.99	rs1942343	T	G	0.86	0.31	0.34	0.28		Pax-5	1.2kb 3' of LINC-ROR
57053547	0.83	0.99	rs1942344	A	G	0.85	0.31	0.34	0.27		Zfp187	1kb 3' of LINC-ROR
57053938	0.85	0.99	rs1942345	G	C	0.86	0.3	0.34	0.28		19 altered motifs	643bp 3' of LINC-ROR
57054639	0.84	0.98	rs948650	G	A	0.86	0.31	0.34	0.28		Gm397	LINC-ROR
57055291	0.85	0.99	rs9949903	A	G	0.86	0.31	0.34	0.28		GR, ZBTB33	LINC-ROR
57055463	0.85	0.99	rs1893445	G	A	0.86	0.31	0.34	0.28		GR	LINC-ROR
57055665	0.82	0.98	rs11334455	AG	A	0.86	0.3	0.34	0.28		10 altered motifs	LINC-ROR
57056163	0.85	0.99	rs6566850	T	C	0.86	0.31	0.34	0.28		5 altered motifs	LINC-ROR
57056177	0.85	0.99	rs6566851	T	A	0.86	0.31	0.34	0.28		4 altered motifs	LINC-ROR
57056519	0.85	0.99	rs8095898	A	C	0.86	0.31	0.34	0.28		NF-kappa B, Spz1	LINC-ROR
57057227	1	1	**rs1942347**	A	T	0.86	0.34	0.4	0.31		**10 altered motifs**	LINC-ROR
57057466	0.86	1	rs1942348	T	C	0.86	0.31	0.34	0.28		NF-E2, NRSF	LINC-ROR
57057964	1	1	rs2027701	A	G	0.86	0.34	0.4	0.31	NFKB, YY1	Egr-1, MIF-1, Pax-4	LINC-ROR
57058041	0.86	1	rs2027702	C	T	0.86	0.31	0.34	0.28	6 bound proteins		LINC-ROR
57064612	0.93	0.98	rs9949774	G	C	0.79	0.34	0.43	0.31		4 altered motifs	LINC-ROR
57069132	0.82	0.92	rs146961366	G	A	0.71	0.32	0.37	0.3		4 altered motifs	LINC-ROR
57074442	0.82	0.91	rs7240941	G	A	0.65	0.44	0.46	0.31			2.3kb 5' of LINC-ROR
57078753	0.86	0.93	rs12103936	A	G	0.84	0.46	0.46	0.31		Foxo, Spz1	6.6kb 5' of LINC-ROR
57079317	0.86	0.93	rs7235435	T	C	0.84	0.46	0.46	0.31		GCM	7.2kb 5' of LINC-ROR
57080624	0.86	0.93	rs12967435	T	C	0.84	0.46	0.46	0.31		Egr-1	8.5kb 5' of LINC-ROR
57083093	0.86	0.93	rs4801080	G	A	0.84	0.46	0.46	0.31		E2F	11kb 5' of LINC-ROR
57084496	0.85	0.92	rs9946685	A	G	0.67	0.44	0.46	0.31			12kb 5' of LINC-ROR
57084832	0.82	0.92	rs33939725	GT	G	0.83	0.46	0.44	0.3		Gm397	13kb 5' of LINC-ROR

pos: position, hg38: human genome release number 38, LD: linkage disequilibrium, Ref: reference allele, Alt: alternative allele, AFR: African, AMR: American, ASN: Asian, EUR: European, freq: frequency. The bold polymorphism is the studied variant in this study. Data source: HaploReg v 4.1. (https://pubs.broadinstitute.org/mammals/haploreg/haploreg.php) (last accessed March 2022).
